# Stakeholder engagement analysis - a bioethics dilemma in patient-targeted intervention: patients with temporomandibular joint disorders

**DOI:** 10.1186/s12967-014-0366-z

**Published:** 2015-01-20

**Authors:** Andre Barkhordarian, Gary Demerjian, Allison Jan, Nateli Sama, Mia Nguyen, Angela Du, Francesco Chiappelli

**Affiliations:** Division of Oral Biology & Medicine, UCLA School of Dentistry, 10833 Le Conte Avenue, CHS 63-090 Los Angeles, CA USA

**Keywords:** Bioethics, Translational science, Temporomandibular joint disorders, Patient-centered outcomes research, Stakeholders engagement analysis, Patient-evidence-provider fit, Patient-targeted intervention

## Abstract

Modern health care in the field of Medicine, Dentistry and Nursing is grounded in fundamental philosophy and epistemology of translational science. Recently in the U.S major national initiatives have been implemented in the hope of closing the gaps that sometimes exist between the two fundamental components of translational science, the translational research and translational effectiveness. Subsequent to these initiatives, many improvements have been made; however, important bioethical issues and limitations do still exist that need to be addressed. One such issue is the stakeholder engagement and its assessment and validation. Federal, state and local organizations such as PCORI and AHRQ concur that the key to a better understanding of the relationship between translational research and translational effectiveness is the assessment of the extent to which stakeholders are actively engaged in the translational process of healthcare. The stakeholder engagement analysis identifies who the stakeholders are, maps their contribution and involvement, evaluates their priorities and opinions, and accesses their current knowledge base. This analysis however requires conceptualization and validation from the bioethics standpoint. Here, we examine the bioethical dilemma of stakeholder engagement analysis in the context of the person-environment fit (PE-fit) theoretical model. This model is an approach to quantifying stakeholder engagement analysis for the design of patient-targeted interventions. In our previous studies of Alzheimer patients, we have developed, validated and used a simple instrument based on the PE-fit model that can be adapted and utilized in a much less studied pathology as a clinical model that has a wide range of symptoms and manifestations, the temporomandibular joint disorders (TMD). The temporomandibular joint (TMJ) is the jaw joint endowed with sensory and motor innervations that project from within the central nervous system and its dysfunction can be manifested systemically in forms of movement disorders, and related pathological symptomatologies.

Currently, there is limited reliable evidence available to fully understand the complexity of the various domains of translational effectiveness, particularly in the context of stakeholder engagement and its assessment, validation as well as the bioethical implications as they pertain to evidence-based, effectivness-focused and patient-centered care.

## Background

The Patient Protection and Affordable Care Act (PPACA-2010) [1] seeks to improve healthcare in the U.S., but it contains many controversial flaws and limitations. The pursuit of translational medicine, dentistry and nursing is based on the fundamentals of translational science, which is composed of two domains, the translational research and the translational effectiveness. Translational research refers to the scientific research the “bench-to-bedside” enterprise of translating knowledge from the basic sciences into the development of new treatments. It is the movement between basic research and patient-oriented research that leads to new or improved scientific understanding. Translational effectiveness refers to translating the findings and results into everyday practice. It facilitates the movement between patient-oriented research and population-based research that leads to better patient outcomes, the implementation of best practices, and improved health status for patients and communities. Together they adhere to the hypothesis-driven scientific process that involves an intimate transaction of the systematic process of patient-centered outcomes research on the molecular and systems pathobiology of the patient, as well as the research synthesis process for the consensus of the best available evidence in patient-targeted interventions. Translational science as a whole aims to increase health literacy of the patients, caregivers and other stakeholders, which in turn demands significant improvements in evidence dissemination empowering them to actively participate in the decision-making process. However, a clear, well defined and bioethically sound procedural methodologies are still missing for assessing, evaluating and analyzing stakeholder engagement. Here, we examine this question in patients with Temporomandibular Joint Disorders (TMD) as a clinical model, because, while this pathology commences as a problem related to the field of dentistry, it can lead to systemic manifestations that involve medicine and nursing. We propose that stakeholder engagement analysis in translational science is bioethically sound, so long as it contributes to the psycho-cognitive, psycho-emotional accommodation and well being of the stakeholder in the reality of the clinical condition at hand [2].

### The temporomandibular joint

The temporomandibular joints are ginglymo-arthrodial bilateral articulations of the mandible with the maxillary bone of the frontal aspect of the skull, with a hinge-type ginglymal and a sliding arthrodial component. Encapsulated by a fibrous tissue, each joint consists of the condylar process of the mandible below, and the glenoid fossa of the temporal bone above. Between these bone surfaces lies the articular meniscal disc, held by tight fibers from below and looser fibers from the temporal bone superiorly, which creates an upper and a lower capsular space. A synovial membrane lines the inner facet of the capsules, and secretes the temporomandibular synovium that fills and lubricates the upper and lower spaces [3]. When adequate support by the relative occlusional position of the upper and lower teeth, the molars in particular, is lacking, the joint is progressively and chronically altered anatomically due to deregulated functions of masticatory muscles, which contributes to temporomandibular joint pathology [3].

The trigeminal nerve, the largest of the twelve cranial nerves is responsible for sensation in the head and neck. Of its three branches, the mandibular branch innervates the lower face and the temporomandibular joint. It projects somatic afferent fibers, and motor innervation to the masticatory musculature (i.e., special visceral efferent fibers) [3]. The trigeminal branches originate from the trigeminal ganglion, whence a single large sensory root enters the brainstem at the level of pons. A number of cranial nerves converge to the pons, besides the mid-pontine sensory and motor trigeminal projections. These include the abducens nerve (cranial nerve VI), which controls the lateral rectus muscle for movement of the eye, the facial nerve (VII), which controls the muscles of facial expression, and the vestibular-cochlear nerve (VIII), which transmits sound and equilibrium (balance) information from the inner ear to the brain. Together they impart to the pons its sensory regulation of hearing, equilibrium and movement, taste, and facial sensation to touch and pain, as well as its motor regulatory function for eye movement, facial expressions, chewing, swallowing, saliva and tear secretion, as well as movement coordination [3,4].

Disorders of the temporomandibular joint encompass a spectrum of diverse conditions, and the term TMD describes a group of conditions with similar signs and symptoms that affect the termporomandibular joints, the muscles of mastication, or both. TMD is often characterized by aching of the muscles of mastication, pain and clicking or popping sounds upon opening and closing the jaw, and severely restricted jaw movement. In our previous studies, we showed that TMD contributes to the development and exacerbation of cervical dystonia and other movement disorders, most likely via neuritis of the auriculotemporal branch of the trigeminal nerve that feeds into the pontine region and controls head and body posture [5]. In those studies we also proposed that the proteomic signature of biomarkers in local (e.g., joint synovial fluid) and distal body fluids (e.g., saliva, cerebrospinal fluid) in a translational research modality would behoove patient-targeted TMD diagnosis and prognosis [6,7]. Our data obtained within the EBD-PBRN also indicated remarkable elevations in certain neuroimmune factors in the synovial fluid of TMD patients that were significantly and positively correlated with diagnostic imaging-rated severity of the TMD condition [8] (Barkhordarian et al., under review) confirming the intimate coexistence of neuroimmunological and neuropathology complexities with TMD.

## Stakeholders in translational medicine

Translational medicine rests on the fundamental principles of stakeholder engagement [2]. Stakeholders are important partners in the clinical decision-making process [2,9,10], particularly in the context of patient-centered healthcare home/neighborhoods for optimizing patient-targeted interventions. However, the evidence on the effectiveness and the bioethics of stakeholder engagement still remains to be limited [11,12].

**Figure 1:**
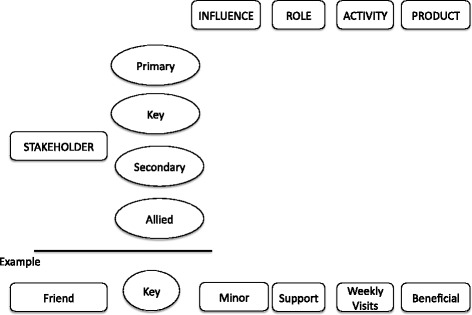
**Stakeholder matrix.** The figure shows a simplified stakeholder matrix to exemplify the process of stakeholder “mapping”. As shown in the example provided in the figure, each stakeholder is identified as primary, key, secondary or allied. The stakeholder’s influence, role activity, and other characteristics are tabulated in the matrix, as well as the stakeholder’s effect and outcome of the healthcare intervention process. In the example provided, we have a “friend”, who could be a secondary stakeholder identified in this case as a key stakeholder, presumably because of possessing a Power of Attorney or directive who makes healthcare decisions on behalf of the patient. We observe the influence of this friend to be, at this present moment, relatively minor as visits are relatively rare. Yet, we recognize beneficial outcomes from those visits (it could also be the case that visits have a seriously detrimental outcome on the patient). This tabulation is prepared for every stakeholder, and is regularly revisited and updated.

Stakeholder theory originally defined stakeholders as those individuals without whose support and feedback an organization, or a project within an organization cannot subsist [13]. In the context of translational medicine, stakeholders are: basic scientists, clinicians/clinical researchers, epidemiologists, health services researchers, patients, family members, caregivers, patient advocates, social workers, insurance carriers, legal advisers. They provide multidimensional contributions to patient-targeted endeavors in evidence-based, effectiveness-focused clinical decision-making processes [8,14]. As shown in Figure 1 stakeholders in modern healthcare engage in translational medicine as:

1. Primary stakeholders: those individuals ultimately and directly affected, either positively or negatively, by the healthcare outcomes (e.g., patients, the immediate family members, caregivers of patients who cannot represent themselves),
2. Key stakeholders: individuals who may or may not be primary stakeholders, but have a significant influence on the decision-making process (e.g., relatives, friends or caregivers empowered by a legal document or directive to make healthcare decisions on behalf of the patient),
3. Secondary stakeholders: individuals indirectly affected by the outcomes, or indirectly involved in the patient’s care process,
4. Allied stakeholders: individuals who are involved in the patient’s care, but are indirectly affected by the healthcare outcome (e.g., medical, dental, nursing and pharmacy staff, other hospital employees, insurance agents, legal staff and lawyers).

The critical questions and issues of bioethics in translational medicine has remained to be addressed in part because individuals who judge themselves to be stakeholders engage *de facto* (in practice but not necessarily ordained by law) as stakeholders raising issues of liability and protection of the confidentiality and security of health care information. Incontrovertible evidence establishes that stakeholder engagement is rarely uniform: not all stakeholders have the same roles and degree of involvement in the healthcare process. Therefore, assessing stakeholder engagement (Table 1) while necessary and critical for translational medicine may be bioethically restricted, biased and compromised. There is a need for concerted research efforts to be directed towards the development and validation of novel analytical tools to establish the nature, level (or quantity), and quality of stakeholder participation in healthcare [15].

**Table 1:** **The seven principal steps of stakeholder analysis**

**Steps**	
**1**	**Defining**: Stakeholders are defined and identified in relation to a specific issue: stakeholder identification operates in respect to a particular specified issue.
**2**	**Long Listing**: A “long list” of key, primary and secondary stakeholders is drawn with respect to the specified issue that indicates groupings (e.g., public, private, and community) and sub-groupings (i.e., gender, ethnicity, age).
**3**	**Mapping**: Analysis of the Long List along selected criteria (i.e., interest, social influence, political role) to allow systematic exploitation of positive attributes, identification of gaps or needed bridge-building among stakeholders are mapped.
**4**	**Visualizing**: Drawing an Influence-Interest-Capacity matrix is essential at this stage.
**5**	**Verifying**: Validity of the analysis is established by assessing and verifying stakeholders availability and commitment. This step may require additional informants and information sources.
**6**	**Mobilizing**: Strategies for sustaining effective participation of the stakeholders, tailored to the different groups and sub-groups of identified stakeholders are mobilized and implemented that includes empowerment interventions for high stake stakeholders with little power or influence.
**7**	**Evaluating**: Reassessment is performed to ensure maximizing the roles and contribution of all stakeholders.

Stakeholder analysis must evolve as systematic, sequential scientific process, subject to a stringent process of construct validation designed to identify and characterize sub-constructs related to stakeholder engagement. Stakeholders’ roles, involvement and function must be quantified, including their (1) actions and position; (2) ability and intent to influence implementation; (3) motivation to participate; and (4) capability to change and adapt as the care situation evolves, and calls for adaptive management [2,16-18]. Reliable tools must be developed, as mentioned, to that end, which must be bioethically coherent and contemporaneously validated to quantify stakeholders’ beneficial input.

In order to assess the reliability of these novel instruments, the analyses of inter-rater reliability and coefficient of agreement [2], as well as generalizability (G) theory validation will be required. The generalizability validation process ensures a flexible approach to simultaneously estimate multiple sources of measurement error variance (i.e., facets), while permitting to generalize the findings of the main analysis across the different study facets. G theory leads to re-calculate the reliability and minimal detectable changes across a variety of combination conditions of these facets. In brief, the G mode of validation of stakeholder engagement analysis will engender the selection of optimal settings minimizing the number of required measures [2,19].

In our preliminary studies (data not included) we have examined the criteria and processes necessary to improve stakeholder engagement by raising their health literacy. We were able to demonstrate that bioethically sound training in communication skills could significantly improve physicians-patient relationship, reduce inappropriate use of medical information, and raise stakeholder engagement [20].

### The Person-Environment (PE) fit theoretical model

To best examine the role and fit of stakeholders in the context of patient-targeted interventions in translational medicine, we considered the well-established and validated theoretical PE fit model. PE-fit establishes the degree to which a person is compatible with the environment, and determines the extent to which someone’s personality and set of skills are adaptable with the environmental demands. It defines the degree to which an individual can successfully adjust to the imperatives of the surrounding. The model arose from the Personality-Job Fit theory, which states in brief that a person’s personality, training and skills determine one’s adaptability and synergy in an organization and a work environment. A perceived (i.e., subjective) or real (i.e., objective) misfit between the person and environment leads to impaired outcomes on specific tasks, job performance, satisfaction and quality of professional and personal life. From an analytical standpoint, the model offers a framework for establishing and predicting how the person’s real and perceived characteristics and skills will fit and adapt to the real and perceived requisites and demands of the work environment. Because the perception of fit of an individual within a given environment is dependent upon one’s ability to discern between perception and reality, the model reveals preventive intervention toward improving the person’s well-being, should it be sub-optimal due to constraining factors in the environment [2,21-23].

The model evolved to address outcomes of ill-health, consequential to the psycho-emotional stress associated with a perceived or real lack of fit, between an individual and the environment. Further evolution rendered the model more widely applicable across a variety of settings in translational medicine, including our study of evidence-based health care for caregivers (i.e., primary stakeholders) of patients with Alzheimer’s disease [24,25].

In that work, we constructed and validated a simple instrument (in-house questionnaire), based on the same criteria described in the literature [21], which consisted of 15 components aimed at quantifying the subjective and objective perceptions of the caregivers and of the immediate demands of the caregiving responsibilities (Table 2) [25]. The data were analyzed along four simple criteria:

**Table 2:** **PE fit for caregivers of patients with Alzheimer’s disease** [25]

1	Overall perception of health	Subjective assessment by the caregiver, overall Fit
2	Perceived energy level	Subjective, caregiver (Person)
3	Perceived mood of patient	Subjective, caregiving responsibilities (Environment)
4	Perceived lifestyle of patient	Subjective, caregiving responsibilities (Environment)
5	Perceived memory of patient	Subjective, caregiving responsibilities (Environment)
6	Perceived family relationships	Subjective, caregiving responsibilities (Environment)
7	Perceived relationship with spouse	Subjective, caregiving responsibilities (Environment)
8	Perceived relationship with friends	Subjective, caregiving responsibilities (Environment)
9	Perceived sense of self	Subjective, caregiver (Person)
10	Ability to perform household chores	Objective, caregiving responsibilities (Environment)
11	Enjoyment of leisure	Objective, caregiving responsibilities (Environment)
12	Ability to hold financial responsibilities	Subjective, caregiver (Person)
13	Perception that own life is ending	Objective, caregiver (Person)
14	Overall life satisfaction	Objective, caregiver (Person)
15	Have intent to hurt self	Objective, caregiving responsibilities (Environment)

- The principal subjective caregiver-environment fit: fit between the subjective perception of the caregiver and his/her perceived responsibilities;
- The secondary objective caregiver-environment fit: fit between the objective abilities of caregiver in the face of the objective demands of the caregiving responsibilities;
- The reality contact: correspondence between the subjective perception of the responsibilities of caregiving and the objective reality posed by these responsibilities; and
- The accuracy, or accessibility, of the self: correspondence between the subjective views and objective reality of the caregiver’ abilities to face these demanding responsibilities.

The subjective person-environment fit (Fs) is the result of the interdependent relationship between the person’s subjective assessment of self (Ps) and his or her subjective evaluation of the environment (Es). In a parallel fashion, the objective environment (Eo) and the objectively assessed person’s abilities to meet its demands (Po) yield a quantification of the objective person-environment fit (Fo). These relationships can be summarized quantitatively as Fo = Eo-Po and Fs = Es-Ps, a can reflect the demand, or need on the part of the person (Np), or the environment (Ne) to actualize fit; and the given abilities of the person (Gp), or the given attributes of the environment (Ge) that facilitates fit. The objective person-environment fit (Fo) is a complex function of the difference (delta, Δ) between the attributes of the environment (Ge) and the need on the part of the person (Np) or the environment (Ne) to actualize fit. In the same vein, subjective person-environment fit (Fs) is a function of the delta between the attributes of the person (Gp) and the need of the environment (Ne) to facilitate fit.

The validity and the reliability of the instrument were tested in 200 subjects stratified, based on clinical exam among the groups of senile dementia of the Alzheimer’s type of stages 1–5 on the Global Assessment Scale (age range: 55–70), and of age-matched non-Alzheimer’s type dementias that included vascular dementias, Parkinson’s dementia, and dementia with Lewy bodies. We also included control subjects with no signs of dementia and of the same age range. Taken together, the inferences derived from this simple instrument provided a critical element to ensure optimal utilization of caregivers in the patient-targeted evidence-based clinical intervention for patients with Alzheimer’s disease [25].

Currently we are in the process of developing a new instrument (questioner) in order to incorporate bioethical issues in the assessment of the stakeholder engagement for patients with TMD. Ongoing studies are validating a revision and expansion of the original instrument, based in part on the ethical dialectics proposed in the literature and above [26]. Some of the bioethical issues that need to be incorporated in the new instrument are highlighted (Table 3).

**Table 3:** **Stakeholder engagement fit general model**

	**Subjective**	**Objective**
Stakeholder	Perception of the stakeholder’s energy level, mood, abilities and skills, and willingness to meet the demands imposed by their engagement in the clinical decision-making process for patient-targeted intervention.	Effectiveness in taking on own role as stakeholder, facing financial hardship, adapting in changing life’s role and routine, and controlling psycho-emotional strain and stress associated with the demands imposed by their engagement in patient-targeted intervention.
Engagement in the clinical decision-making process	Stakeholder’s understanding and knowledge of the clinical condition at hand, of the options for treatment, of the best available evidence (i.e., health literacy).	***Bioethical concerns***, including privacy, bias, ensuring “individualized equal care” [27], minimizing “cumulative incremental risks” [28], and values [29].

To further establish the applicability of the PE fit approach to bioethical concerns in translational medicine, we propose the person-environment fit model to a patient-evidence-provider best-fit model that is grounded on the same principles outlined above [2]. In this more complex approach, eight primary outcomes were identified (below) to describe the relation among objective and subjective patients, evidence and providers. These domains are used in order to develop specific criteria for the stakeholder engagement assessment tool.

1. Patient objective – Evidence objective – Provider Objective: optimal translational medicine
2. Patient objective – Evidence objective – Provider Subjective: sub-optimal translational medicine, with potential bias on the part of the provider
3. Patient objective – Evidence subjective – Provider subjective: health care based on the evidence, with added concern of potentially biased provider
4. Patient Objective – Evidence subjective – Provider objective: health care based on the evidence, provider unbiased
5. Patient subjective – Evidence Objective – Provider subjective: although the best available evidence is used, caveat of bias due subjective patient and provider
6. Patient subjective – Evidence subjective – Provider subjective: evidence-based of the evidence, with all possible drawbacks of subjective assessments
7. Patient subjective – Evidence objective – Provider objective: attempt is made for translational medicine, but patient is biased with pre-conceived notions: provider must help patient “un”learn the misguided information before the best available evidence can be given
8. Patient subjective – Evidence subjective – Provider objective: provider needs to be taught how to gain access to the best available evidence in order to carry out translational medicine effectively [2].

## Conclusion

Primary areas of bioethical concern subsist in the context of patient-targeted intervention in translational medicine. They are: (1) selection bias in the sampling process, (2) invasiveness of the screening and treatment protocols, (3) infraction to the fundamental principles of human rights and human dignity, and (4) reliable process of research synthesis for obtaining the best available evidence for patient-targeted intervention, and unbiased dissemination and utilization in specific clinical settings. The latter pertains to the domain of translational effectiveness, which we have discussed previously [30], and involves the issues of stakeholder engagement assessment and analysis we have considered here, in line, with the need for “individualized equal care” proposed by others [27]. Incontestable compelling evidence shows that from a bioethical standpoint, patients and their representatives have the right to be included in decisions that pertain to patient-targeted interventions. It is also self-evident that this privilege cannot be extended to all stakeholders, and that stakeholder engagement analysis is needed to contribute positive impact on the transparency and accountability of translational science. Therefore, reaching a shared understanding of ethical roles and responsibilities amongst stakeholders require dialogue and reciprocal awareness of limitations.

## Abbreviations

AHRQ: Agency for Healthcare Research Quality
EBD-PBRN: Evidence-Based Decisions Practice-Based Research Network
PE fit: Person-Environment fit
PPACA: Patient Protection and Affordable Care Act
PCORI: Patient Centered Outcomes Research Institute
NIH: National Institutes of Health
TMD: Temporomandibular joint disorders
